# Diffusion kernel-based predictive modeling of KRAS dependency in KRAS wild type cancer cell lines

**DOI:** 10.1038/s41540-021-00211-8

**Published:** 2022-01-19

**Authors:** Bastian Ulmer, Margarete Odenthal, Reinhard Buettner, Wilfried Roth, Michael Kloth

**Affiliations:** 1 Institute of Pathology, Cologne University Hospital, Cologne, Germany; 2 Institute of Pathology, Mainz University Hospital, Mainz, Germany

**Keywords:** Cancer, Computational biology and bioinformatics, Molecular biology

## Abstract

Recent progress in clinical development of KRAS inhibitors has raised interest in predicting the tumor dependency on frequently mutated RAS-pathway oncogenes. However, even without such activating mutations, RAS proteins represent core components in signal integration of several membrane-bound kinases. This raises the question of applications of specific inhibitors independent from the mutational status. Here, we examined CRISPR/RNAi data from over 700 cancer cell lines and identified a subset of cell lines without KRAS gain-of-function mutations (KRAS^wt^) which are dependent on KRAS expression. Combining machine learning-based modeling and whole transcriptome data with prior variable selection through protein-protein interaction network analysis by a diffusion kernel successfully predicted KRAS dependency in the KRAS^wt^ subgroup and in all investigated cancer cell lines. In contrast, modeling by RAS activating events (RAE) or previously published RAS RNA-signatures did not provide reliable results, highlighting the heterogeneous distribution of RAE in KRAS^wt^ cell lines and the importance of methodological references for expression signature modeling. Furthermore, we show that predictors of KRAS^wt^ models contain non-substitutable information signals, indicating a KRAS dependency phenotype in the KRAS^wt^ subgroup. Our data suggest that KRAS dependent cancers harboring KRAS wild type status could be targeted by directed therapeutic approaches. RNA-based machine learning models could help in identifying responsive and non-responsive tumors.

## Introduction

The RAS signaling pathway is a key driver of carcinogenesis in many different tumor entities^1–4^. Frequently, gain-of-function mutations or copy number alterations (CNA) at different levels of the signaling cascade lead to overactivity and thereby increased cell growth, migration, and invasion^2,3,5^. Due to its high relevance in cancer per se, intensive research has been conducted aiming to develop targeted therapies. In recent years, several drugs entered clinical application focusing on inhibitors that are directed against membrane receptors preventing constitutively activated signal transduction^6–8^. EGFR represents one of the most well-known examples^9^. However, therapy is limited by various resistance mechanisms in the receptor itself or other RAS/RAF pathway elements^7,10–12^. Common mechanisms with high clinical relevance include mutations in downstream RAS GTPases^2,3,10^, which represent important nodes in signal integration from cell membrane to nucleus^13^. Here, somatic point mutations lead to constitutive activation downstream of membrane receptors, thereby hindering therapeutic success^5,10^. Despite intensive research, drug binding pockets could not be identified in these proteins for a long time, making the development of direct inhibitory pharmacotherapy difficult. MEK inhibitors have become a first option to overcome this mechanism of resistance by inhibiting downstream mitogen-activated protein kinase kinases. However, until now, clinical efficacy has only been demonstrated for specific applications such as NRAS/BRAF mutated melanoma^14–16^. More recently, mutation-specific and panKRAS inhibitors have been developed that inhibit KRAS activity directly or indirectly^17–20^. The mutation-specific inhibitors exploit structural changes in the KRAS protein that result from oncogenic point mutations so that cells expressing wild-type protein are less affected, which is expected to reduce toxicity of the therapy. However, so far specific inhibitors could only be designed for a few KRAS mutations such as G12C. This led to the development of the panKRAS inhibitors, which downregulate KRAS activity by binding to SOS1 and thus enable an application independent of the mutation status. PanKRAS inhibitors such as BI1701963 are currently being tested in clinical trials including combinations with MEK inhibitors^21^. Furthermore, following the results of a phase II trial, Sotorasib became the first drug of mutation-specific inhibitors to receive preliminary approval for patients with therapy-refractory NSCLC^22^.

The emergence of new therapies with small molecule RAS inhibitors also increases the relevance of identifying responsive and resistant tumors as accurately as possible. Depending on the drug and tumor entity, different markers are currently used as predictors. Those include mutations, CNA and gene expression^6,23^. The selection of predictors depends on the respective tumor entity and its characteristics. For example, activating EGFR and ERBB2 mutations are considered positive predictive markers for therapy with tyrosine kinase inhibitors against receptors of the EGFR-family in non-small cell lung cancer (EGFR) and colorectal cancer (ERBB2)^23,24^. Response to ERBB2-antibody Trastuzumab correlates with ERBB2 expression and copy number status in breast cancer^6,25^. Furthermore, EGFR resistance mutations such as T790M are important in anti-EGFR therapy in non-small-cell lung cancer as well as activating KRAS mutations in colorectal cancer^11,26^. Besides activating mutations, expression-based RAS signatures may also improve therapy response prediction including treatment with KRAS inhibitors^27,28^. This could be particularly important in tumors with more complex activation mechanisms by yet unknown RAS-activating events (RAE).

**Fig. 1:**
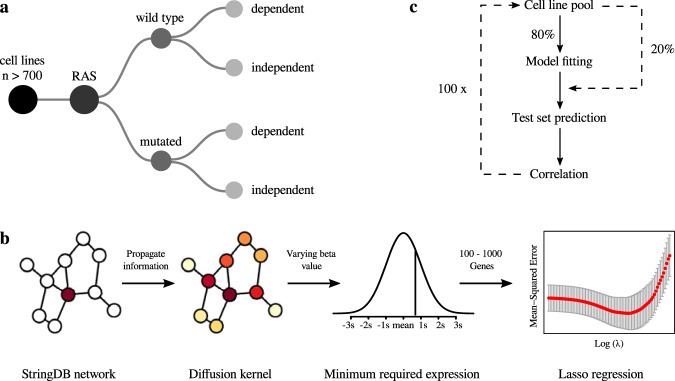
Cancer cell line classification algorithm and gene dependency modeling strategies. **a** Strategy of cell line subgrouping leading to the investigated subgroups (HRAS^wt^/HRAS^mut^, KRAS^wt^/KRAS^mut^, NRAS^wt^/NRAS^mut^). **b** Variable selection workflow for whole transcriptome RNA-expression data consisting of the construction of a literature-based gene network followed by further selections steps with centrality quantification through a diffusion kernel and a minimum required expression level. Several different constellations of the hyperparameters were tested. Final modeling was performed using a Lasso, Elastic Net or Random Forest regression. **c** Workflow of iterative model fitting and performance evaluation for each gene dependency dataset.

Recent progress in clinical development of KRAS inhibitors has raised interest in predicting the tumor dependency on frequently mutated RAS-pathway oncogenes. However, even without such activating mutations, RAS proteins represent core components in signal integration of several membrane-bound kinases. This raises the question of applications of the inhibitors independent from the mutational status. In this work, we analyzed KRAS dependency by CRISPR/RNAi data from the Achilles- and DRIVE-Project^29–32^. We identified a subgroup of KRAS dependent cell lines harboring wild type status in KRAS (Fig. 1a). KRAS dependency of this subgroup could not be predicted by RAE-based models or those of previously published expression signatures. Instead, our machine learning approach based on whole transcriptome data and diffusion kernel-based variable selection using protein-protein interaction network analysis significantly improved KRAS dependency prediction in KRAS^wt^ cancer cell lines (Fig. 1b/c, see methods).

## Results

### Characterization of KRAS dependency in KRAS wild type cancer cell lines

**Fig. 2:**
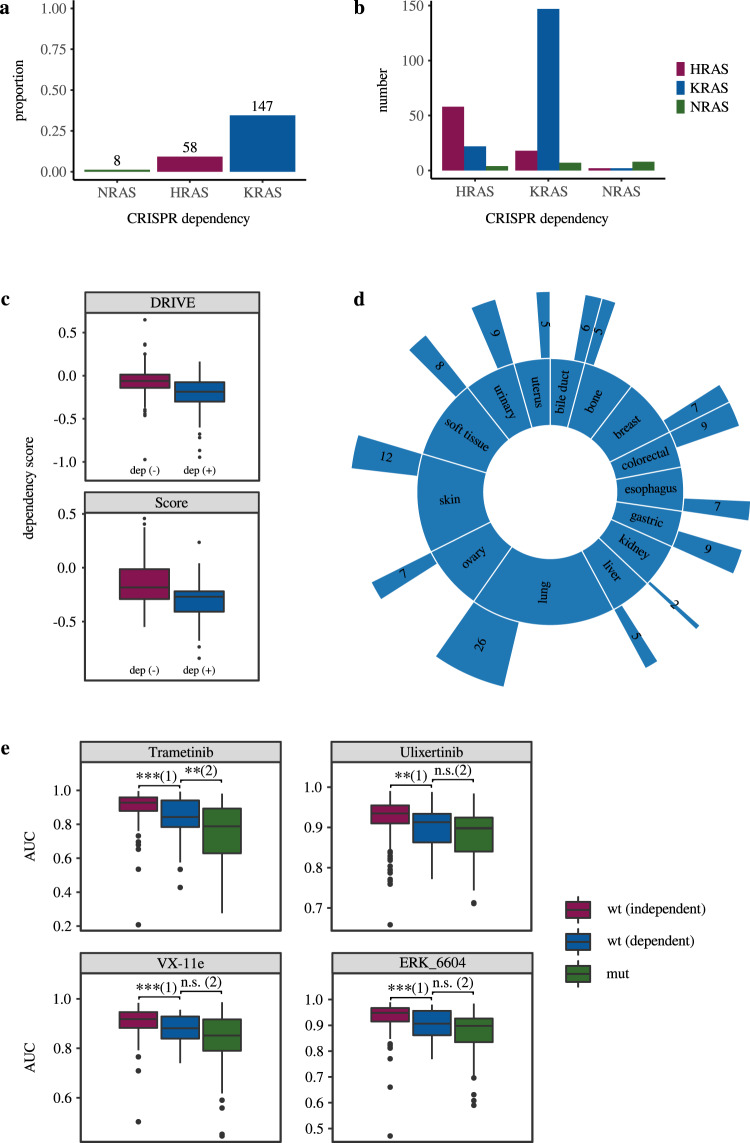
RAS dependency characterization and associations to MEK inhibitor responsivity. **a** Proportion of gene-dependent cell lines in the four subgroups of wild type cancer cell lines. The numbers above the columns indicate the absolute number of dependent cell lines. **b** Total number of co-dependencies for each gene. **c** Validation of the elaborated KRAS dependent and independent subgroups. After dividing the cell lines into KRAS dependent and independent using the Achilles Project CRISPR data, we verified the existence of the two subgroups in data of the DRIVE (RNAi) and the Score Project (CRISPR). Cell lines classified as KRAS dependent in the Achilles data exhibited a significantly higher dependency in both screens (Wilcox Test; DRIVE: *p* = 4,6 * 10^−10^, *n* = 342; Score: *p* = 2.1 * 10^−4^, *n* = 124). **d** Proportions of the different entities in the KRAS^wt^ group (inner circle) and the proportion of KRAS-dependent cell lines in each entity with indicated absolute numbers (outer circle). Only entities with at least ten cell lines were included in the figure. In absolute numbers, lung tumors were the most represented entity among KRAS dependent cell lines, followed by skin tumors. Overall, the group is very heterogeneous without one entity clearly dominating. **e** Characterization of MEK-inhibitors sensitivity in KRAS^wt^ cancer cell lines with dependent (blue, wt (dependent)) independent (purple, wt (independent)) status and as a reference KRAS^mut^ cases (green, mut). The symbols above the brackets refer to the following significance codes: *** < 0.001; ** < 0.01; * < 0.05]; ‘n.s.’ > 0.05. In the overall comparison between the three groups, KRAS^wt^ cell lines with KRAS dependency are significantly more responsive to MEK inhibitors (lower AUC) than KRAS^wt^ independent group, but for some inhibitors less responsive than the KRAS^mut^ cell lines (Wilcox Test: Trametinib: (1) *p* = 4.6 * 10^−4^ (*n* = 183), (2) *p* = 7.4 * 10^−3^ (*n* = 113); Ulixertinib: (1) *p* = 4.1 * 10^−3^ (*n* = 183), (2) *p* = 9.5 * 10^−2^ (*n* = 112); VX-11e: (1) *p* = 4.9 * 10^−4^ (*n* = 178), (2) *p* = 9.7 * 10^−2^ (*n* = 111); ERK_6604: (1) *p* = 1.8 * 10^−4^ (*n* = 179), (2) *p* = 1.3 * 10^−1^ (*n* = 111)). For further compounds of CCLE, GDSC1, and GDSC2 see also Supplementary Fig. 1. Box plot annotation (**c**, **e**): 25th percentile (box bottom), 75th percentile (box top), median (box center), whiskers top/bottom ±1.5 × interquartile range, outliers are shown as dots.

Currently, several KRAS inhibitors are being tested in clinical trials, first preliminary approvals have been granted and further are likely to follow^20–22,33^. Primarily these inhibitors are developed with the aim to overcome gain-of-function mutations^17–20^. However, there is evidence that patients with wild type status in RAS/RAS-oncogenes may also benefit from therapies targeting RAS genes^28^. To investigate this phenomenon, we analyzed CRISPR knockout data of cancer cell lines from the Achilles Project focusing on a dependency characterization of KRAS, NRAS, and HRAS^29–31,34^. These data provide a valid approximation of chemosensitivity to inhibitors targeting wild type RAS, which are not yet available in databases such as GDSC or CCLE. The KRAS^wt^ subgroup exhibited the largest fraction of dependent cell lines followed by HRAS^wt^ (Fig. 2a), whereas dependencies in NRAS^wt^ tended to be limited to a few cases. Co-dependencies of the individual wild type subgroups, i.e., the simultaneous presence of two dependencies, were most frequently observed for KRAS^wt^ and HRAS^wt^, but rather rare overall (Fig. 2b). To validate the existence of a KRAS dependent subgroup in KRAS^wt^ cell lines we additionally analyzed two other dependency data sources of the DRIVE- (RNAi) and the Score-Project (CRISPR). Again, cell lines classified as KRAS dependent in the initial Achilles CRISPR screen exhibited a significantly higher dependency in both data (Fig. 2c). Next, we examined the data for an association between tissue origin and KRAS dependency to rule out any potential bias. For each entity, we performed a Fisher test with the respective binary characteristics of belonging to the individual entity (yes/no) and KRAS dependency (yes/no). Although we observed a proportionally increased number of KRAS-dependent cases for colorectal and gastric cell lines within the entities (Fisher test; colorectal: *p* = 0.03; gastric: *p* = 0.03; *n* = 573), the overall composition of the dependent cell lines demonstrated a heterogeneous distribution (Fig. 2d). With 26 cases, the largest fraction of responsive cell lines originated from the lung, followed by skin tumors with 12 and tumors of the central nervous system (CNS) with 11 cases. However, these entities account for only 18% (lung), 9% (skin), and 8% (CNS) of the subgroup. We therefore assumed only a limited impact of tissue-specific effects on the results of our further studies.

Our results so far suggest that KRAS has an important survival function in signal integration in a specific subgroup of KRAS^wt^ cell lines. To further investigate a potential clinical relevance, we analyzed drug sensitivity to several downstream interacting MEK inhibitors. For this analysis, we additionally excluded cell lines with non-deleterious mutations in BRAF, HRAS, and NRAS from the KRAS^wt^ subgroup (RAS^wt^/RAF^wt^). We chose AUC (GDSC) or Active Area (CCLE) for drug sensitivity quantification because validity of extrapolated IC50 values is limited for cell lines that were only partially responsive or unresponsive within the experimentally tested inhibitor concentrations, as stated by GDSC^35^. To this end, the AUC has been successfully used in a variety of other publications^36–38^. As expected, we found consistent associations to MEK inhibitors across multiple compounds and datasets (Fig. 2e, Supplementary Fig. 1). Overall, the sensitivity of the KRAS^wt^ cell lines with KRAS dependency was significantly higher compared to the KRAS^wt^ independent subgroup, but slightly lower than the one of the KRAS^mut^ cell lines. Thus, we were able to provide further evidence for an increased RAS activity in the depicted subgroup. Furthermore, these analyses suggest that a combined therapy approach with panKRAS and MEK inhibitors, as it is currently being tested in KRAS mutated tumors^21^, may also be effective for patients with RAS^wt^/RAF^wt^ tumors.

### RAE possess limited sensitivity and specificity for KRAS dependency prediction in KRAS^wt^ subgroup

**Fig. 3:**
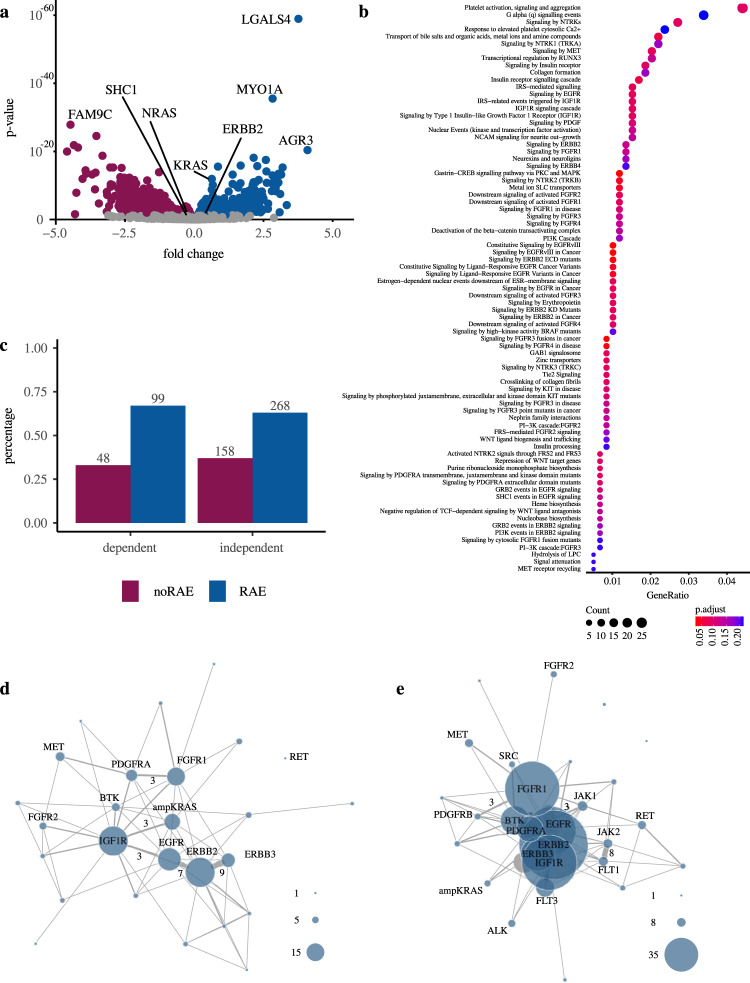
Transcriptional characterization and dependency analysis of KRAS dependent KRAS^wt^ cell lines. **a** Differentially expressed genes in KRAS^wt^ dependent vs independent cell lines (*n* = 567). Positive values on the *x*-axis reflect higher expression in the dependent subgroup, correspondingly negative values reflect higher expression in the independent subgroup. **b** Overrepresentation analysis (Reactome) of genome wide CRISPR screen genes exhibiting a higher dependency in the KRAS^wt^ dependent subgroup (Wilcoxon–Mann–Whitney Test; *n*_genes_ = 1038). **c** Percentage of cell lines harboring at least one RAE (blue) or no RAE (purple) in KRAS^wt^ subgroup for KRAS dependent (left) and independent (right) cell lines. Absolute values are shown above each column. **d**, **e** Binary co-dependency network of RAE in KRAS^wt^ highlighting the heterogeneous distribution of RAE (Dependent cell lines (**d**), independent (**e**)). Number of co-dependencies shared between two genes is shown if there were more than two co-dependencies. Node size refers to the number of cell lines classified as dependent on the respective gene. Cell lines without RAE were not included in the figures.

According to our results, KRAS^wt^ cancers with KRAS dependency potentially reflect a subgroup of cancers with therapeutic relevance. Therefore, we aimed to specify this subgroup in more detail. There are numerous well characterized mechanisms of RAS activation that can lead to KRAS dependency (RAS activating events, RAE). These include mutations, overexpression and CNA in upstream genes as well as amplification of KRAS itself^6,23,39–41^. Using differential expression analysis in KRAS^wt^ cancer cell lines between KRAS dependent and independent subsets we were able to detect several of already known pathway activating regulators. These included ERBB2 and KRAS (Fig. 3a), which were significantly overexpressed in the KRAS dependent KRAS^wt^ subgroup. In addition, overrepresentation analysis revealed an association of the differentially expressed genes (*n*_genes_ = 358) to various receptor tyrosine kinase (RTK) signaling pathways such as EGFR, FGFR, MET, NTRK, and IGFR (Supplementary Data 1). However, RAE identification based on molecular genetic events or expression is not trivial and may lead to erroneous conclusions. Mutations provide an illustrative example, as their clinical implications are sometimes not known with certainty^42–44^.

Therefore, we used the genome wide CRISPR data from the Achilles Project for the detection of RAE, as they reflect direct information about the survival potential of the gene. Consistent with the expression data, genes of the pathways mentioned above were associated with higher dependency in the KRAS dependent subgroup. In addition, we found other RTK pathway associations such as those to KIT and PI3K (Fig. 3b). In the next step, we analyzed each cell line for potential RAE that could be causal for KRAS dependency. In total we used 43 binary markers (Supplementary Data 2) including 42 gene dependencies and KRAS amplification status, as this is associated with worse outcome and tumor progression in different cancer types^40,45,46^ (see methods). This simple approach allowed us to assign at least one RAE to 67% of the KRAS dependent cell lines either by co-dependency or amplification (Fig. 3c). For the remaining 33%, other monogenic activation mechanisms that were not considered or complex-genetic activation would be conceivable. In addition, our analysis showed that the distribution of markers is relatively heterogeneous and that multiple co-dependencies may also occur (Fig. 3d/e). However, a causal relationship is not assured by this approach. Eventually, independent simultaneous occurrences cannot be ruled out despite targeted and literature-based marker selection. Furthermore, it may not always be accurate to conclude that an RAE leads to KRAS dependency. Strikingly, this is illustrated by repeating the analysis on wild type cell lines without KRAS dependency, which assigned a RAE to 63% of the cell lines (Fig. 3c). To further reveal more complex interactions between RAE and KRAS dependency, we performed Lasso regression using quantitative RAE dependencies as predictors (see methods). This first approach based on RAE achieved no significant correlation between model predictions and experimental data (Pearson’s *r* = 0, *p* = 0.92, *n* = 529), which underlines the need for improved variable selection and integrative modeling.

### KRAS dependency prediction in KRAS^wt^ subgroup using previously published RNA signatures

Our initial approach indicates some challenges in the prediction of KRAS dependency in KRAS^wt^ cancers by known RAE. We therefore searched for suitable alternative modeling strategies. Regarding the identification of RAS- and KRAS-dependent cancers, several approaches have been taken including RNA-based expression signatures^27,28^. To characterize whether these known signatures represent an improvement in prediction, we created machine learning models and examined their predictive performance (see methods). Overall, the resulting predictive performance was not satisfactory. Models based on the signature published by Loboda et al. achieved a correlation of 0.15 (Pearson’s *r*, *n* = 567), those based on the signature by Singh et al. 0.18 (Pearson’s *r*, *n* = 567). However, it should be noted that we were not able to assign all gene identifiers in the given signatures, leading to a loss of four genes (2.7%) in the signature by Loboda et al. and 14 (2.7%) in the signatures from Singh et al. (Supplementary Data 3). Due to the small number of missing genes, we considered the influence on the results to be neglectable in both cases. Consequently, our analyses indicate a more complex situation in the prediction of KRAS dependency in KRAS^wt^ cancers even with previously published expression signatures.

### Diffusion kernel-based protein-protein interaction network analysis improves predictive modeling of KRAS dependency in KRAS^wt^ cancer cell lines

**Fig. 4:**
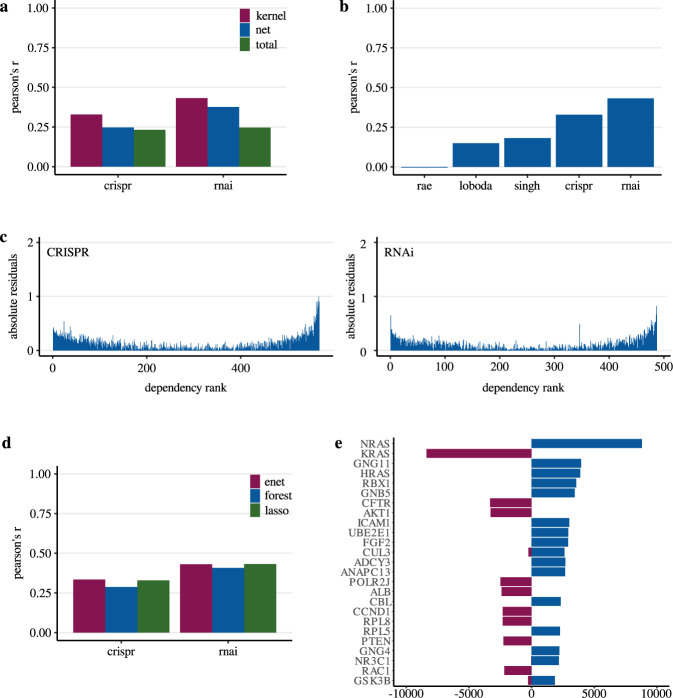
Performance of different KRAS dependency modeling strategies and predictor analysis in KRAS^wt^ cell lines. **a** Correlation analysis (Pearson’s r) in independent test sets between the experimentally determined KRAS cancer cell line dependency and our machine learning-based predictions for varying sets of predictors (see methods). Results are shown for models using all available predictors of the RNA sequencing data (total), all available predictors of the protein interaction network (net) and predictors selected by the diffusion kernel with hyperparameter optimization (kernel). Models were based on KRAS^wt^ cell lines of the different datasets (crispr - Achilles CRISPR effect data (*n* = 567), rnai - DRIVE RNAi (DEMETER2) data (*n* = 487)). In case of the diffusion kernel variable selection workflow maximum correlation was reached with a hyperparameter constellation using 500 predictors. For complete results of hyperparameter tuning see Supplementary Data 4. **b** Performance (Pearson’s r) of KRAS dependency models in KRAS^wt^ group compared between the different approaches (RAE – RAE-based models (CRISPR data), Loboda – Models using RNA expression of the gene selection by Loboda et al. (CRISPR data), Singh – Models using RNA expression of the gene selection genes by Singh et al. (CRISPR data), CRISPR – Best performing models using RNA expression of the gene selection by the diffusion kernel with optimized hyperparameters (CRISPR data), RNAi – Best performing models using RNA expression of the gene selection by the diffusion kernel with optimized hyperparameters (RNAi data)). For CRISPR/RNAi correlation analysis was performed similarly to (**a**). Correlation coefficients for RAE, Loboda and Singh were determined as described above. **c** Absolute error of CRISPR/RNAi models for each cell line using mutation- and best performing RNA-predictor set. Summarized results of 400 unique models are shown in the two waterfall plots. Cell lines were ordered by ascending observed KRAS dependency from left to right. The absolute error was estimated by summing the individual absolute differences of the predicted values from the observed values. **d** Correlation analysis (Pearson’s r, *n*_CRISPR_ = 567, *n*_RNAi_ = 487) performed similarly to (**a**) this time comparing models using different algorithms (Elastic Net regression - enet, Random Forest regression – forest, Lasso regression - lasso). Neither Elastic net nor Random Forest Regression could improve the Lasso predictions of KRAS dependency. **e** Occurrence frequency of RNA-predictors in 12000 unique models of KRAS dependency (CRISPR/RNAi) in KRAS^wt^ cancer cell lines. Only models using the variable selection by the diffusion kernel were included. Negative values indicate the frequency of how often the predictor had a negative coefficient in the models (associated with higher KRAS dependency), positive values the frequency of how often the predictor had a positive coefficient (associated with lower KRAS dependency). The 25 most redundant genes are shown here.

Consistent with our results so far, recent literature showed that gene dependencies of KRAS, NRAS, and HRAS are difficult to model by whole transcriptome analysis and that mutation status is proposed to be more robust^47^. To improve modeling in KRAS^wt^ cancers, we tested our own strategies including different machine learning algorithms as well as variable selection through gene centrality estimation in a protein-protein interaction network by a diffusion kernel (See methods, Fig. 1b/c). Initially, reference models with all predictors available in the RNA expression dataset were created (47768 genes) using Lasso regression. Compared to the previous signatures we achieved significantly higher correlations between test set predictions and the experimentally determined dependency demonstrating that the predictive performance of RNA-based models can be improved depending on the modeling strategy. For the CRISPR data Pearson’s r was 0.23 and for RNAi data 0.25 (Fig. 4a). However, due to the high number of predictors, it is possible that the models are prone to overfitting. Dataset-specific artifacts such as random associations of predictors to the dependent variable, characteristics of the cell line model or effects caused by the entity distribution could affect external validity. To further improve the performance and to test strategies against possible overfitting we included a variable selection step before modeling. This strategy involved an initial restriction of predictors to genes derived from a literature-based protein-protein interaction network (StringDB), followed by further reduction steps through estimation of gene centrality in the network using a diffusion kernel (See methods, Fig. 1b/c). The hypothesis is that the selected genes are important regulatory elements in KRAS signaling and thus represent suitable predictors of KRAS dependency with biological significance. This approach combined with additional hyperparameter optimization (see methods) resulted in a total of 105 different predictor sets with a size ranging from 100 to 1000 genes. In the subsequent modeling by Lasso regression with optimized hyperparameters we reached a maximum correlation of 0.43 (Pearson’s r) between observed and predicted KRAS dependency (Fig. 4a, Supplementary Data 4). This corresponds to a performance improvement of 72% compared to the reference models with all available predictors of the RNA sequencing data. Best predictions could be achieved by using an initial variable set reduced to only 500 genes. Additional models based on the complete gene set of the protein network consistently performed weaker when compared to the diffusion kernel-based prediction (Fig. 4a).

These results demonstrate that variable selection by the network-based approach significantly improves the performance of models predicting KRAS dependency in KRAS wild-type cancer cell lines. The predictions were clearly superior to RAE-based models or those of previously published expression signatures (Fig. 4b). In addition, it enables a substantial reduction of predictors prior modeling without impairing model performance. However, a closer analysis of the models revealed specific challenges. For all models, we observed that absolute errors increase significantly toward the outsides of the distribution, thereby also reflecting outliers which are difficult to predict (Fig. 4c). We assumed biological effects or attributes of dependency distribution to be the cause for this observation. Therefore, we tested Elastic Net and Random Forest regression as two additional algorithms for modeling. However, these methods could not improve the prediction accuracies (Fig. 4d). For the predictor sets selected by the diffusion kernel maximum observed correlation coefficients (Pearson’s r) with Elastic Net regression after hyperparameter optimization were 0.33 (CRISPR data) and 0.43 (RNAi data). Interestingly, despite the skewed distribution of the KRAS dependency with more independet cell lines the decision tree-based Random Forest regression performed worse than the linear regression models (Pearson’s r; CRISPR: 0.29; RNAi: 0.41).

### Central components of KRAS interaction network contain non-substitutable information signals for dependency modeling

Next, we characterized the predictors of KRAS dependency in KRAS wild type models. Among the 12,000 models of CRISPR/RNAi data using gene sets selected by the diffusion kernel and hyperparameter optimization, the frequency of non-zero model coefficients was quantified for each predictor. In total, 1964 genes were used at least once in a model. Most frequently used predictors were NRAS and KRAS expression, followed by GNG11, HRAS, and RBX1 (Fig. 4e, Supplementary Data 5). KRAS expression was associated with an increase, NRAS, GNG11, HRAS, and RBX1 with a decrease of KRAS dependency. The inverse regulation of KRAS, NRAS, and HRAS expression in context of KRAS dependency is consistent with results from Fig. 2b showing that co-dependency between these genes is a rare event in KRAS wild type cancer cell lines. We also performed an overrepresentation analysis of predictors that were used in at least 10% of all models, which corresponds to a list of 63 genes. As expected, numerous pathways related to signal transduction were overrepresented because of the variable selection based on the KRAS centralized network (Supplementary Data 6). Accordingly, the results show a profile comparable to Fig. 3b. Most significant regulated pathways were extra-nuclear estrogen signaling (*p* = 3.1 * 10^−16^), Diseases of signal transduction by growth factor receptors and second messengers (*p* = 2.4 * 10^−15^) as well as several pathways which involve tyrosine kinase signaling. Again, this highlights the heterogeneity of RAE and further potential approaches for combination therapies in the KRAS^wt^ subgroup.

The results indicate that KRAS dependent cancer cell lines in the KRAS^wt^ group exhibit a distinctive phenotype or activation state, which might be represented by expression data. However, RNA expression data are highly structured and intercorrelated^31,48^, so that genes selected by our literature-based approach might be replaced without losing predictive power of the models. Consequently, a specific phenotype or gene regulation in the context of KRAS dependency would be less likely. On the other hand, a decrease in prediction power would mean a loss of information that could not be compensated by other predictors and context-specific regulation would be more likely. To investigate this, we selected the models with best performing hyperparameters and extracted all predictors with non-zero coefficients, resulting in lists of 293 (CRISPR) and 184 (RNAi) genes. Then, separately for each gene list and dependency dataset we excluded the lists from the superset of all proteins in the interaction network and repeated modeling with these selections. In fact, we found a lower correlation for both datasets compared to the models using the total number of network genes. Performance in models of Achilles CRISPR dependency data dropped about 20% (Pearson’s *r* = 0.20, *n* = 567) and for DRIVE RNAi data about 29% (Pearson’s *r* = 0.27, *n* = 487). The loss of predictive power suggests that a non-compensable loss of information has taken place. This speaks for a regulation of genes selected by our interaction network-based in context of KRAS dependency, indicating a KRAS dependency phenotype in the KRAS^wt^ subgroup.

### Comparison of RNA expression data and KRAS mutation status as predictors of generalized KRAS dependency

**Fig. 5:**
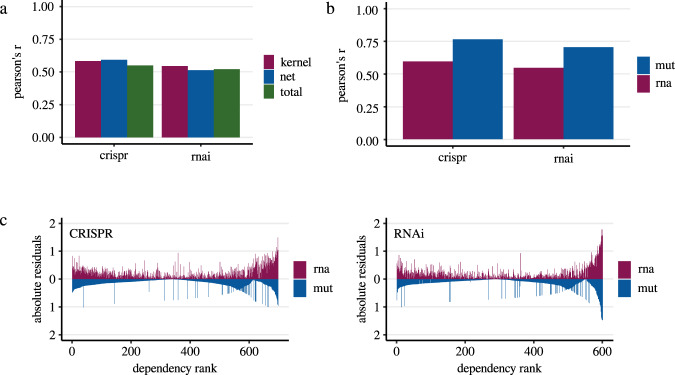
Performance of mutation status-/RNA expression-based KRAS dependency models and analysis of error distributions in the complete cell line dataset. **a** Correlation analysis (Pearson’s *r*) in independent test sets between the experimentally determined KRAS cancer cell line dependency and our machine learning-based predictions for varying sets of predictors (see methods). Results are shown for models using all available predictors of the RNA sequencing data (total), all available predictors of the protein interaction network (net) and predictors selected by the diffusion kernel with hyperparameter optimization (kernel). For both dependency datasets models were based on the entire cell line set (crispr - Achilles CRISPR effect data (*n* = 698), rnai - DRIVE RNAi (DEMETER2) data (*n* = 601). In case of diffusion kernel variable selection workflow maximum correlation was reached with a hyperparameter constellation using 1000 predictors. For complete results of hyperparameter tuning see Supplementary Data 4. **b** Correlation analysis (Pearson’s *r*) performed similarly to (**a**) this time comparing models either using KRAS mutation status (mut) or best performing predictors of the RNA sequencing data (rna) in the respective datasets (crispr - Achilles CRISPR effect data (*n*_rna_ = 698, *n*_mut_ = 704), rnai - DRIVE RNAi (DEMETER2) data (*n*_rna_ = 601, *n*_mut_ = 613). Using RNA sequencing data as predictors, the best performance was achieved either with the complete protein interaction network (CRISPR) or a subset of the network consisting of 1000 genes selected by the diffusion kernel (RNAi). **c** Absolute error of CRISPR/RNAi models for each cell line using mutation- and best performing RNA-predictor set (CRISPR: complete protein interaction network; RNAi: diffusion kernel selection with 1000 genes). Summarized results of 400 unique models are shown in the two waterfall plots. Cell lines were ordered by ascending observed KRAS dependency from left to right. The absolute error was estimated by summing the individual absolute differences of the predicted values from the observed values (RNA expression-based (rna): purple bars; Mutation status-based (mut): blue bars). Predictions of models using mutation status show two local minima in the absolute error distributions indicating the binary prediction results. For both types of predictors residuals tend to increase at both sides of the distribution.

Finally, after predicting KRAS dependency in the KRAS^wt^ subgroup we also applied our selective modeling strategy to the entire set of cancer cell lines. Initial reference models using Lasso regression and all predictors available in the RNA expression dataset achieved correlations (Pearson’s r) of 0.55 (CRISPR) and 0.52 (RNAi) between observed and predicted KRAS dependency (CRISPR) (Fig. 5a). Prior variable selection by the diffusion kernel yields slight improvements in performance (Pearson’s r: 0.58 (CRISPR), 0.54 (RNAi)). Using all available predictors of the protein interaction network, a minimal improvement in prediction accuracy was observed for the CRISPR data again (Pearson’s *r*: 0.59), but not for the RNAi data, where models performed weaker (Pearson’s *r*: 0.51). Despite the good results, consistent with recently published literature models based on KRAS mutation status outperformed RNA-based models (Fig. 5b; Pearson’s *r*: 0.76 CRISPR, 0.7 RNAi)^47^. Again a closer analysis of the models revealed specific challenges. For both types of predictors, we observed that absolute errors increase significantly toward the outsides of the distribution (Fig. 5c). As expected, the mutation-based models provided near-binary predictions, as they were built on three binary predictors which cannot represent the continuous distribution of KRAS dependency. This is illustrated by the distribution of absolute residuals in Fig. 5c, which exhibits two local minima in the mutation-based models, each reflecting one of the two almost constant prediction values of the models. In contrast, RNA-based models did allow quantitative prediction and identified potential borderline cases. Nevertheless, as before, they struggled to adequately predict highly responsive cases. These results show distinct advantages and disadvantages of the two types of predictors. Accordingly, comprehensive models of KRAS dependency may benefit from a combination of KRAS mutation status and RNA expression data, as well as other predictor types.

## Discussion

The RAS signaling pathway possesses a central position in the oncogenesis of several tumor entities via numerous mechanisms. Recent progress in clinical development of KRAS inhibitors has raised interest in predicting the tumor dependency on frequently mutated RAS-pathway oncogenes. However, even without such activating mutations, RAS proteins represent core components in signal integration of several membrane-bound kinases. This raises the question of applications of the inhibitors independent from the mutational status.

In this work we identified a KRAS dependent subset of KRAS wild type cell lines. Consistently, across six different datasets from CRISPR, RNAi, and chemosensitivity experiments, we found evidence for increased RAS activity in the elaborated subgroup. Our results suggest that patients without activating KRAS mutations may also benefit from targeted therapies against KRAS. Here, compounds such as the SOS1-KRAS interaction inhibitors BI-3406 or BI-1701963 could be of particular interest. In contrast to mutation-specific inhibitors^17^, these pan-KRAS inhibitors do not require a specific mutation and could be used in the therapy of KRAS wild type malignancies^19,20^. In addition, since the subgroup exhibits a significantly increased sensitivity to MEK inhibitors, a combined therapy, as currently being tested in clinical trials for KRAS mutated tumors, may also offer further advantages for some patients^21^.

Our results provide evidence for the presence of a KRAS-dependent subgroup in KRAS^wt^ tumors. The high number of KRAS-dependent cell lines within the KRAS^wt^ subgroup raises expectations for an equally high proportion of patients with increased response to therapy. In the future, further clinical research efforts are needed. In this regard our study should serve for deeper understanding of mechanisms in panKRAS inhibition and associated current clinical trials.

Identification of responsive and resistant tumors will be an essential task for an optimal therapy of the identified subgroup. We demonstrated that RAEs such as KRAS amplifications or EGFR co-dependencies are not reliable predictors of KRAS dependency in the KRAS^wt^ subgroup. Moreover, RAEs were heterogeneously distributed, and presence of RAE did not necessarily follow a KRAS dependency and vice versa. This observation is corroborated by results from clinical trial data, as the presence of an activating KRAS G12C mutation predicted response to Sotorasib in only 32% of patients^17^. We interpreted this as further evidence for the complexity of signal transduction as previously described^9,49^. To address this complexity, we investigated modeling solutions using whole transcriptome RNA sequencing data as predictors. Recently, difficulties in RNA-based modeling of gene dependencies of the tumor drivers KRAS, NRAS, and HRAS have been described^47^.

In our analyses we tested modeling using Lasso regression with varying sets of predictors with a focus on variables selected by a literature-based protein-protein interaction network and subsequent gene centrality quantification through a diffusion kernel. This systematic approach with an initial variable reduction to 100–1000 genes significantly improved predictions of KRAS dependency in KRAS^wt^ cell lines when compared to models using all available RNA predictors, RAE-based models or those of previously published expression signatures. Although not the main focus of the depicted study, we also achieved improvements in performance by our approach in the complete cell line set but could not outperform KRAS mutation status.

The importance of variable selection for modeling is emphasized by inferior predictions of models based on genes of previous RAS expression signatures. In the respective initial publication these signatures could provide robust predictions for training and test data^27,28^. However, in our study the reported performance dropped significantly. This suggests that predictions of expression-based models are only valid for applications with high similarity to the training data and tend to overfitting. Generalizations of specific gene signatures without methodological and algorithmic reference seem to be limited, which underlines the need for standardized procedures for the prediction of therapy response to KRAS inhibitors in tumor patients.

Even with high agreement between the training set data and those of the planned application, steps to reduce overfitting caused by artifacts in the training data are highly relevant to ensure external validity. In this regard, besides the use of statistical methods suitable for high-dimensional datasets collecting additional information about the predictors to select variables with context specific biological relevance can be important before modeling. Literature-based protein-protein interaction networks as used in this work represent one option for this purpose since they enable the integration of preexisting scientific knowledge to identify key regulatory genes in the context of KRAS signaling. These genes possibly represent more robust predictors, which reduce overfitting caused by dataset-specific artifacts. Due to the lack of additional data, we were not able to validate this hypothesis. Nevertheless, the significantly improved internal performance of KRAS dependency models in the KRAS^wt^ subgroup, using predictors selected by the diffusion kernel, indicate the advantages of this strategy. Future efforts with the objective to establish diagnostically applicable models may possibly benefit from similar approaches as well.

In summary, our results suggest that a subset of patients without oncogenic KRAS mutations may benefit from targeted therapy with KRAS inhibitors. In the long run, we assume that machine learning models based on high-dimensional RNA expression data could help with therapeutic decisions. As already mentioned before, a crucial factor for clinical applicability of the proposed models will be a highly standardized test methodology. This includes all steps of the analysis including sample preparation, sequencing and bioinformatic analysis as well as the choice of a suitable parameter for therapy response quantification. The realization of such solutions appears to be possible and reasonable as costs for quantifying gene expression continue to fall.

## Methods

### Data

Cancer cell line data were obtained from the website of Dependency Map Consortium including Genomic, RNA-expression, CRISPR, RNAi and CCLE drug sensitivity data (Release 21Q1)^29–32,34,50^. GDSC drug data (Release 8.2) were downloaded from the project’s website^51^. For all analyses, only cell lines originating from solid tumors were used.

### Cell line classification

Cell line classification was performed in two steps using mutation and CRISPR dependency data of the Achilles project. The investigated entire cell line set was divided into wild type and mutated according to their mutation status in the examined CRISPR knockout gene. All cell lines harboring non-deleterious mutations in KRAS, NRAS, and HRAS were regarded as mutated, reflecting typically activating hotspot mutations. This resulted in six different groups of cell lines (HRAS^wt^/HRAS^mut^, KRAS^wt^/KRAS^mut^, NRAS^wt^/NRAS^mut^). Initial analyses were performed in cell lines with wild type status and for each gene individually (Fig. 1a). In a second step, each group was divided into dependent and independent cell lines by the respective CRISPR gene dependency using the Achilles dependency format. The format indicates the probability of being part of a distribution of essential or non-essential genes for each cell line and each gene (Supplementary Fig. 2). At a threshold of over 50%, cell lines were classified as dependent.

### Binary RAE classification

For RAE classification based on CRISPR dependency we selected oncogenes of the oncoKB database with a restriction to receptor kinases and non-RTKs annotated in HGNC^52–54^. After that we searched for co-dependencies between the selected genes and KRAS using Achilles CRISPR dependency format. As before, cell lines were classified as dependent if dependency exceeded a threshold of 50%. RAE detection based on KRAS amplification status was performed using CCLE copy number data. Cell lines were classified as KRAS amplified if the relative copy number of KRAS exceeded a threshold of 3 compared to the mean copy number of the sample.

### Overrepresentation analysis

Overrepresentation analysis was performed using R package ReactomePA^55^. Following differential expression analysis, consideration was given to all statistically significant negative and positive associations with a minimum required level of gene expression higher than the 75th percentile. For CRISPR data, only those with significantly increased dependency in the KRAS-dependent group (Wilcoxon–Mann–Whitney test) were used.

### Dependency modeling

CRISPR/RNAi data of Achilles and DRIVE projects (gene effect format [−∞; ∞]) were used as dependent variables in gene dependency modeling. Different predictor types (RAE, mutations, RNA expression) were processed as follows. RAE were represented by Achilles CRISPR gene effect format and KRAS gene copy number. We restricted gene dependency RAE to oncogenes annotated in oncoKB database and receptor kinases/non-RTKs from HGNC^52–54^, resulting in 43 different markers for RAE-based predictions (Supplementary Data 2). Using a similar approach to Dempster et al., mutations were divided into three categories (deleterious, hotspot, other) based on annotations from DepMap data^47^. The categories hotspot and other were restricted to non-silent, non-deleterious mutations with or without TCGA/COSMIC hotspot classification. Subsequently, a binary predictor variable was created from each of the three categories. In the presence of one or more mutations from one of the categories, the respective cell line was classified as mutated in the corresponding predictor variable, and as non-mutated in the absence of mutations in the category. Gene identifiers of previous expression signatures (Supplementary Data 3) were obtained from the respective publications and were used without further selection steps^27,28^. To quantify gene expression levels, we used CCLE RNA sequencing data in TPM format.

For our workflow based on whole transcriptome RNA-expression data (TPM) we either used the total number of available RNA predictors or subsets selected by our variable selection approach. For this purpose, we constructed a literature-based protein-protein network to identify significant predictors by gene centrality (Fig. 1b). The hypothesis is that these genes are important regulatory elements in KRAS signaling and thus represent suitable predictors of KRAS dependency. First, protein-protein interactions were downloaded from the STRING database^56^. Gene identifiers were assigned to each protein and duplicated interaction scores between the same genes were averaged. Interactions with a score lower than the 90th percentile were discarded. To focus the network on genes which may be involved in the context of KRAS signaling we restricted genes to those with a direct KRAS interaction (1st shell) and their respective interaction partners (2nd shell). The final network consisted of 7070 genes. Centrality (closeness) of each gene was determined by a diffusion kernel which captures the information flow within the network as previously described^57,58^. [Eq. 1]

1 $${{{{{\mathrm{K}}}}}} = {{{{{\mathrm{e}}}}}}^{\beta {{{{{\mathrm{H}}}}}}} = {{{{{\mathrm{I}}}}}} + \beta {{{{{\mathrm{H}}}}}} + \frac{{\beta ^2}}{{2!}}{{{{{\mathrm{H}}}}}}^2 + \frac{{\beta ^3}}{{3!}}{{{{{\mathrm{H}}}}}}^3 + ...$$

K refers to the diffusion kernel, e to Euler’s number, H to the negative Laplacian matrix, I to the identity matrix and *β* to a parameter which controls the degree of information flow. Similar to Lee et al.^58^ we tested several values of the *β* parameter (*β* = 0,75^n^, *n* = 1, 5, 10, 20, 30) for variable selection. This resulted in seven different closeness estimates of the genes in our network.

Hyperparameter optimization of the variable selection by the diffusion kernel was performed for the following variables. The minimum level of gene expression was tested for the percentiles P25, P50, P75, and P95. *β* parameter was tuned for the values mentioned above. Subsequently, the number of selected genes by centrality was tested for 100, 500, and 1000 genes. This approach resulted in a total of 60 different predictor sets for modeling. Independent of the hyperparameter optimization, we also tested modeling either with all unfiltered genes of the network (6967 genes) or with the total number of RNA predictors available (47,768 genes). For all predictor sets gene expressions with missing values were discarded before the final selection step.

Despite our variable selection workflow, the number of predictors for RNA-based models was still high. To prevent overfitting, we consistently used Lasso regression for all models and predictor types. In addition, we also tested Elastic Net and Random Forest regression in KRAS dependency models of the KRAS^wt^ subgroup after variable selection through the diffusion kernel. Lasso and Elastic Net regression minimize the following quantities [Eqs. 2–3]:

2 $$\beta _{{{{{{\mathrm{Lasso}}}}}}} = \mathop {\sum}\limits_{{{{{{\mathrm{i}}}}}} = 1}^n {\left( {y_{{{{{\mathrm{i}}}}}} - \beta _0 - \mathop {\sum}\limits_{j = 1}^p {\beta _jx_{{{{{{\mathrm{i}}}}}}j}} } \right)} ^2 + \lambda \mathop {\sum}\limits_{j = 1}^p {\left| {\beta _j} \right|}$$

3 $$\beta _{{{{{{\mathrm{Elastic}}}}}}\;{{{{{\mathrm{net}}}}}}} = \mathop {\sum}\limits_{{{{{{\mathrm{i}}}}}} = 1}^n {\left( {y_{{{{{\mathrm{i}}}}}} - \beta _0 - \mathop {\sum}\limits_{j = 1}^p {\beta _jx_{{{{{{\mathrm{i}}}}}}j}} } \right)} ^2 + \lambda \mathop {\sum}\limits_{j = 1}^p {\beta _j^2} + \lambda \mathop {\sum}\limits_{j = 1}^p {\left| {\beta _j} \right|}$$

*n* is the number of observations, *p* the number of different predictors, *β*_0_ the *y*-intercept, *β*_*j*_ the coefficient of the respective predictor, *y*_i_ the values of the independent variable, and *x*_i*j*_ the values of the predictors.

For every set of independent variables 100 models were trained, each using 80% of the cell lines randomly selected by R base sample() function. The remaining 20% served as an independent test set. Test set predictions were averaged for each predictor set and correlated with the experimental data (Fig. 1c).

### Statistical analyses

All statistical analyses were performed in R programming language^59^. The package glmnet was used for Lasso regression and Elastic Net^60^. Random Forest regression was performed with randomForest package^61^. Differential expression analyses were carried out with DESeq2^62^ and network analysis was generated with igraph^63^. RAWGraphs 2.0 was used for additional graphics^64^. Standard statistical methods are mentioned in figures and legends.

### Reporting summary

Further information on research design is available in the Nature Research Reporting Summary linked to this article.

## Supplementary information

Supplementary Information

Reporting Summary

Supplementary Data 1-6

## Data Availability

The datasets analyzed in the study are available on the websites of the Dependency Map Consortium (https://depmap.org/portal/) and the Genomics of Drug Sensitivity in Cancer Project (https://www.cancerrxgene.org/).

## Code availability

All computational analyses were performed in R programming language^59^. The codes used for the other computational analyses are available upon request to the authors.
