# Supplementary material for: Diffusion kernel-based predictive modeling of KRAS dependency in KRAS wild type cancer cell lines
Source: NPJ Syst Biol Appl. 2022 Jan 19;8:2. doi: 10.1038/s41540-021-00211-8 (PMC8770632; doi:10.1038/s41540-021-00211-8)
Supplement: Supplementary file 1 — Supplementary Information [file 41540_2021_211_MOESM1_ESM.pdf]

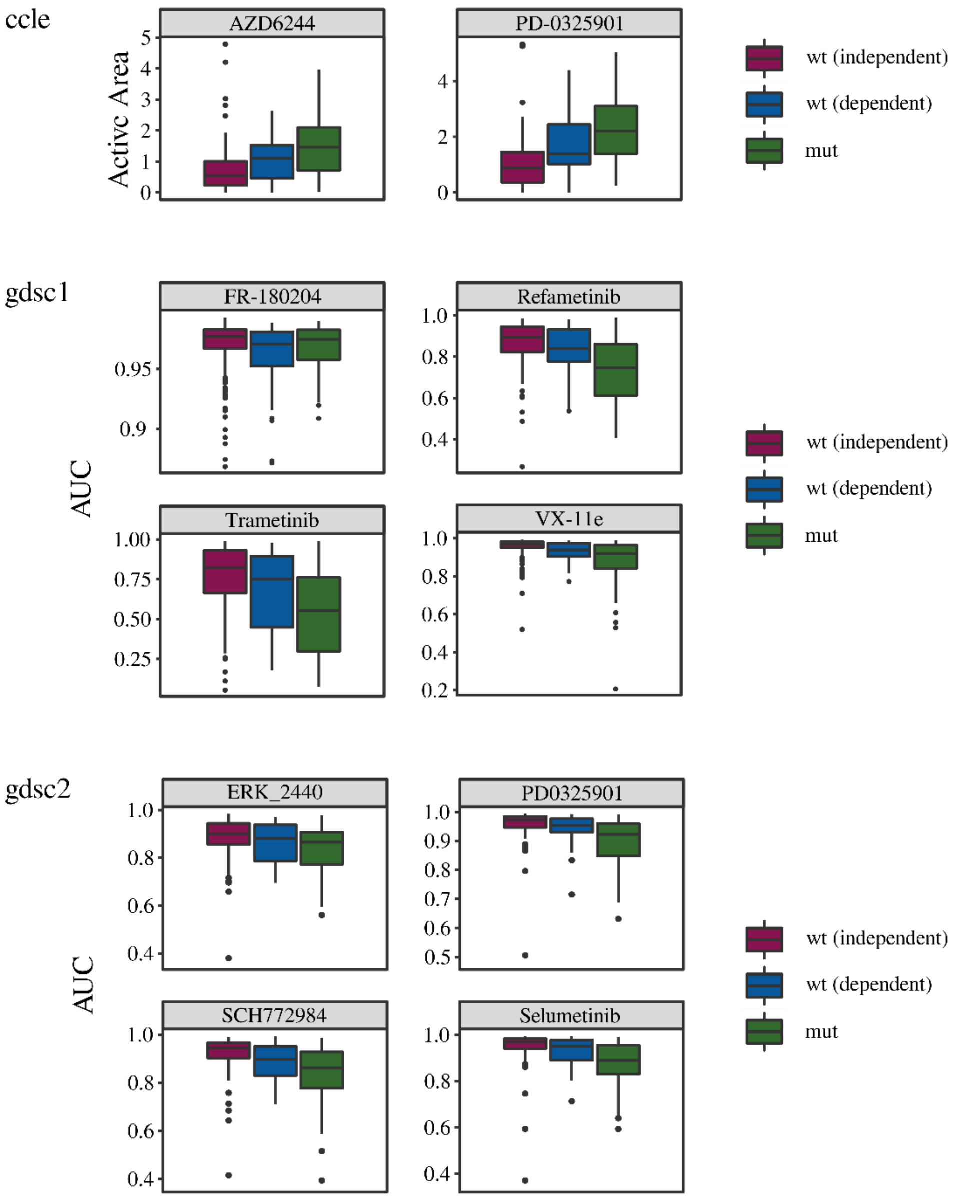

Supplementary Figure 1: Further characterization of MEK-inhibitors sensitivity KRAS^{wt} cancer cell lines dependent on KRAS expression marked with blue (wt (dependent)) and independent of KRAS expression marked with purple (wt (independent)). As a reference we also added KRAS^{mut} cases by a third box plot (green, mut). For CCLE data active area and for GDSC data AUC was used to compare drug responsivity. All compounds show a significantly higher responsivity in the KRAS dependent wild type subgroup compared to the KRAS independent group (Wilcox Test: wild type independent vs dependent (1), wild type dependent vs mutated (2); CCLE: AZD6244 (1) p=3.4 * 10^{-3} (n=155), (2) p=1.1 * 10^{-2} (n=212); PD-0325901 (1) p=1.2 * 10^{-4} (n=155), (2) p=7.6 * 10^{-3} (n=212); GDSC1: Trametinib (1) p=1.7 * 10^{-2} (n=201), (2) p=3.2 * 10^{-3} (n=120); Refametinib (1) p=2.4 * 10^{-2} (n=213), (2) p=5.1 * 10^{-4} (n=128); FR-180204 (1) p=2.3 * 10^{-2} (n=210), (2) p=1.9 * 10^{-1} (n=124); VX-11e (1) p=1.3 * 10^{-3} (n=210), (2) p=6.2 * 10^{-2} (n=124); GDSC2: ERK_2440 (1) p=3.5 * 10^{-2} (n=179), (2) p=1.6 * 10^{-1} (n=111); PD0325901 (1) p=1.5 * 10^{-2} (n=188), (2) p=3.0 * 10^{-4} (n=117); SCH772984 (1) p=3.9 * 10^{-4} (n=187), (2) p=4.7 * 10^{-2} (n=117); Selumetinib (1) p=8.5 * 10^{-3} (n=179), (2) p=4.4 * 10^{-3} (n=111)). Box plot annotation: 25th percentile (box bottom), 75th percentile (box top), median (box center), whiskers top/bottom +/- 1.5 x interquartile range, outliers are shown as dots.

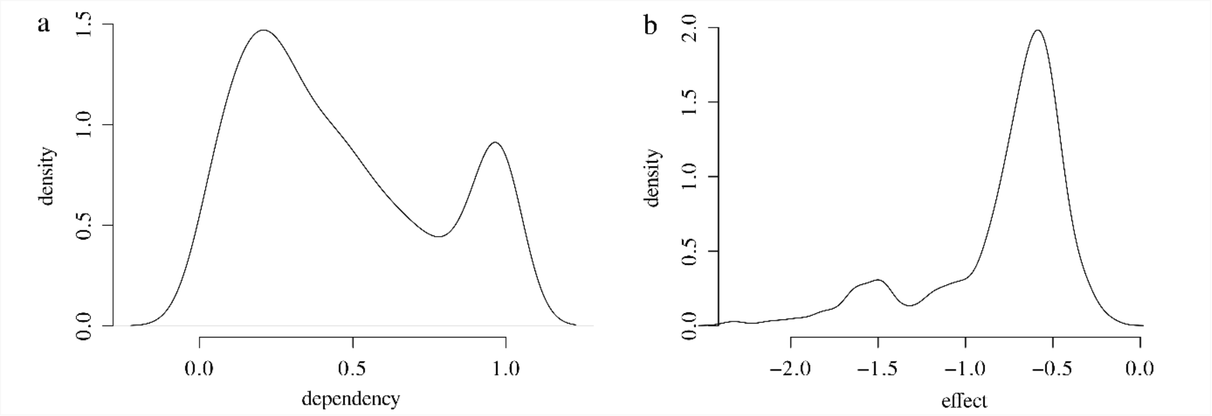

Supplementary Figure 2: Density plots of KRAS dependency distribution (a) Achilles CRISPR dependency format. (b) Achilles CRISPR effect format. The range of the dependency format is restricted to values between 0 and 1 indicating the probability of the gene being part of a distribution of essential or non-essential genes. Achilles effect format and DRIVE RNAi (DEMETER2) data we used for linear modeling have no such restrictions.
