# Supplementary material for: Diffusion kernel-based predictive modeling of KRAS dependency in KRAS wild type cancer cell lines
Source: NPJ Syst Biol Appl. 2022 Jan 19;8:2. doi: 10.1038/s41540-021-00211-8 (PMC8770632; doi:10.1038/s41540-021-00211-8)
Supplement: Supplementary file 2 — Reporting Summary [file 41540_2021_211_MOESM2_ESM.pdf]

nature portfolio

Corresponding author(s): *Double-blind peer review submissions: write DBPR and your manuscript number here instead of author names.*

Last updated by author(s): Nov 11, 2021

Reporting Summary

Nature Portfolio wishes to improve the reproducibility of the work that we publish. This form provides structure for consistency and transparency in reporting. For further information on Nature Portfolio policies, see our Editorial Policies and the Editorial Policy Checklist.

Statistics

For all statistical analyses, confirm that the following items are present in the figure legend, table legend, main text, or Methods section.

n/a	Confirmed	
☐	☒	The exact sample size (*n*) for each experimental group/condition, given as a discrete number and unit of measurement
☐	☒	A statement on whether measurements were taken from distinct samples or whether the same sample was measured repeatedly
☐	☒	The statistical test(s) used AND whether they are one- or two-sided<br>*Only common tests should be described solely by name; describe more complex techniques in the Methods section.*
☐	☒	A description of all covariates tested
☐	☒	A description of any assumptions or corrections, such as tests of normality and adjustment for multiple comparisons
☐	☒	A full description of the statistical parameters including central tendency (e.g. means) or other basic estimates (e.g. regression coefficient) AND variation (e.g. standard deviation) or associated estimates of uncertainty (e.g. confidence intervals)
☐	☒	For null hypothesis testing, the test statistic (e.g. *F*, *t*, *r*) with confidence intervals, effect sizes, degrees of freedom and *P* value noted<br>*Give P values as exact values whenever suitable.*
☐	☒	For Bayesian analysis, information on the choice of priors and Markov chain Monte Carlo settings
☐	☒	For hierarchical and complex designs, identification of the appropriate level for tests and full reporting of outcomes
☐	☒	Estimates of effect sizes (e.g. Cohen's *d*, Pearson's *r*), indicating how they were calculated

Our web collection on statistics for biologists contains articles on many of the points above.

Software and code

Policy information about availability of computer code

Data collection	Cancer cell line data were obtained from the website of Dependency Map Consortium including Genomic, RNA-expression, CRISPR, RNAi and CCLE drug sensitivity data (Release 21Q1). GDSC drug data (Release 8.2) were downloaded from the project's website.
Data analysis	All statistical analyses were performed in R programming language (Version 4.1.1). The package glmnet was used for Lasso and Elastic Net regression (Version 4.1-2). Random Forest regression was performed with randomForest package (Verision 4.6-14). Differential expression analyses were carried out with DESeq2 (Version 1.32.0), overrepresentation analyses with ReactomePA (Version 1.36.0) and network analysis was generated with igraph (Version 1.2.6). RAWGraphs 2.0 was used for additional graphics.

For manuscripts utilizing custom algorithms or software that are central to the research but not yet described in published literature, software must be made available to editors and reviewers. We strongly encourage code deposition in a community repository (e.g. GitHub). See the Nature Portfolio guidelines for submitting code & software for further information.

Data

Policy information about availability of data

All manuscripts must include a data availability statement. This statement should provide the following information, where applicable:

- Accession codes, unique identifiers, or web links for publicly available datasets
- A description of any restrictions on data availability
- For clinical datasets or third party data, please ensure that the statement adheres to our policy

All cancer cell line data are available on the website of Dependency Map Consortium (https://depmap.org/portal/) including Genomic, RNA-expression, CRISPR, RNAi and CCLE drug sensitivity data (Release 21Q1). GDSC drug data (Release 8.2) are available on the project's website (https://www.cancerrxgene.org/).

Field-specific reporting

Please select the one below that is the best fit for your research. If you are not sure, read the appropriate sections before making your selection.

☒ Life sciences ☐ Behavioural & social sciences ☐ Ecological, evolutionary & environmental sciences

For a reference copy of the document with all sections, see nature.com/documents/nr-reporting-summary-flat.pdf

Life sciences study design

All studies must disclose on these points even when the disclosure is negative.

Sample size	All publicly available data of solid tumor cell lines tested in the different screens have been integrated into our analyses.
Data exclusions	Because of biological and therapeutic differences, we have restricted our analyses to solid tumors in order to limit the heterogeneity of our collective. Furthermore, the newly developed KRAS inhibitors are currently tested exclusively in solid tumors, so that an application in the near future is most likely to be expected here.
Replication	The data for our analyses come from various publicly available data sets. Replicates of the measurements were performed as part of these studies.
Randomization	Cell lines were classified based on the investigated characteristics (KRAS mutation status/dependency). For the separation into training and test set during the modeling process, we used the R sample() function for random assignment.
Blinding	Blinding was not possible because we performed a retrospective analysis on data from cell culture experiments.

Reporting for specific materials, systems and methods

We require information from authors about some types of materials, experimental systems and methods used in many studies. Here, indicate whether each material, system or method listed is relevant to your study. If you are not sure if a list item applies to your research, read the appropriate section before selecting a response.

Materials & experimental systems

n/a	Involved in the study	
☒	☐	Antibodies
☐	☒	Eukaryotic cell lines
☒	☐	Palaeontology and archaeology
☒	☐	Animals and other organisms
☒	☐	Human research participants
☒	☐	Clinical data
☒	☐	Dual use research of concern

Methods

n/a	Involved in the study	
☒	☐	ChIP-seq
☒	☐	Flow cytometry
☒	☐	MRI-based neuroimaging

Eukaryotic cell lines

Policy information about cell lines

Cell line source(s)	All cancer cell line data are available on the website of Dependency Map Consortium (https://depmap.org/portal/) including Genomic, RNA-expression, CRISPR, RNAi and CCLE drug sensitivity data (Release 21Q1). GDSC drug data (Release 8.2) are available on the project's website (https://www.cancerrxgene.org/).
Authentication	We performed a retrospective analysis of publicly available cell line data, so no additional authentication was performed from our side.
Mycoplasma contamination	We performed a retrospective analysis of publicly available cell line data, so no testing for Mycoplasma could be performed by our site.
Commonly misidentified lines (See ICLAC register)	Since we used a variety of different cell lines in our analyses, single misidentifications within the data sets cannot be ruled out. However, due to the high quality studies from which the data are derived, we assume only a very small proportion.
